# Effect of a Cognitive Training Program on the Platelet APP Ratio in Patients with Alzheimer’s Disease

**DOI:** 10.3390/ijms21145110

**Published:** 2020-07-20

**Authors:** Tiziana Casoli, Cinzia Giuli, Marta Balietti, Paolo Fabbietti, Fiorenzo Conti

**Affiliations:** 1 Center for Neurobiology of Aging, IRCCS INRCA, 60121 Ancona, Italy; m.balietti@inrca.it (M.B.); fiorenzo.conti@staff.univpm.it (F.C.); 2 Geriatrics Operative Unit, IRCCS INRCA, 63023 Fermo, Italy; c.giuli@inrca.it; 3 Unit of Geriatric Pharmacoepidemiology, IRCCS INRCA, 87100 Cosenza, Italy; p.fabbietti@inrca.it; 4 Department of Experimental and Clinical Medicine, Section of Neuroscience and Cell Biology, Università Politecnica delle Marche, 60126 Ancona, Italy

**Keywords:** Alzheimer’s disease, platelets, APP, cognitive training, biomarkers, synaptic plasticity

## Abstract

In patients with Alzheimer’s disease (AD), synaptic plasticity seems to be involved in cognitive improvement induced by cognitive training. The platelet amyloid precursor protein (APP) ratio (APPr), i.e., the ratio between two APP isoforms, may be a useful peripheral biomarker to investigate synaptic plasticity pathways. This study evaluates the changes in neuropsychological/cognitive performance and APPr induced by cognitive training in AD patients participating in the “My Mind Project”. Neuropsychological/cognitive variables and APPr were evaluated in the trained group (*n* = 28) before a two-month experimental protocol, immediately after its termination at follow-up 1 (FU1), after 6 months at follow-up 2 (FU2), and after 24 months at follow-up 3 (FU3). The control group (*n* = 31) received general psychoeducational training for two months. Some memory and attention parameters were significantly improved in trained vs. control patients at FU1 and FU2 compared to baseline (Δ values). At FU3, APPr and Mini Mental State Examination (MMSE) scores decreased in trained patients. Δ APPr correlated significantly with the Δ scores of (i) MMSE at FU1, (ii) the prose memory test at FU2, and (iii) Instrumental Activities of Daily Living (IADL), the semantic word fluency test, Clinical Dementia Rating (CDR), and the attentive matrices test at FU3. Our data demonstrate that the platelet APPr correlates with key clinical variables, thereby proving that it may be a reliable biomarker of brain function in AD patients.

## 1. Introduction

Over the past few years, a growing number of studies showed that cognitive stimulation, cognitive training, and cognitive rehabilitation programs improve the cognitive state of dementia patients [1,2,3]. This is interesting given that current pharmacological treatments provide only temporary relief of clinical symptoms and are unable to alter disease progression [4]. In particular, the effects of such programs on patients with Alzheimer’s disease (AD) depend on a variety of factors, such as type of intervention, dementia stage, and individual cognitive reserve [5,6]. For instance, cognitive stimulation significantly improves global cognition in individuals with mild-to-moderate dementia but does not seem to affect mood, activities of daily living, or challenging behavior [7,8]. Preclinical studies of animal models and human investigations employing neuroimaging measures suggested that the effects induced by cognitive interventions rely on synaptic plasticity [9,10,11,12,13]; in particular, they first induce functional changes, whereas structural changes in the relevant cognitive pathways appear at a later stage [14,15]. Peripheral blood biomarkers capable of reflecting changes in the pathways involved in synaptic plasticity could provide a fast and informative approach to assess treatment responses and predict outcomes. Amyloid precursor protein (APP), a transmembrane protein highly expressed in neurons and platelets, could be such a biomarker. APP has cell adhesion properties, is involved in central and peripheral synaptogenesis, and its cleavage products, such as 38 to 43 amino acid amyloid-β peptides, secreted APP (sAPP), CTF83, and CTF99, affect synaptic function and strength [16,17]. APP overexpression and knockout both impair synaptic plasticity in animal models [18,19], with several studies describing putative roles for it in the modulation of neurite outgrowth and synaptic connectivity [20,21,22]. Peripheral platelets are the primary APP source in the general circulation [23], with their APP levels comparable to those found in the brain [24]. In Western blot studies, the platelet APP ratio (APPr), i.e., the ratio between the upper 120–130 kDa and the lower 110 kDa isoforms, was found to be significantly lower in AD patients than in healthy controls [25,26,27]. Notably, APPr correlates with Mini Mental State Examination (MMSE) scores and was successfully applied to monitor the effects of acetylcholinesterase inhibitors donepezil and galantamine in AD patients [28,29,30]. In the present study, the platelet APPr was evaluated in AD patients participating in the ‘‘My Mind Project”, which investigates the effects of a comprehensive cognitive training program on cognitive performances and peripheral blood biomarkers [31,32,33,34,35], to test the hypothesis that it reflects central changes induced by the intervention and that such changes correlate with cognitive and/or neuropsychological test scores.

## 2. Results

### 2.1. Baseline Assessment

**Figure 1:**
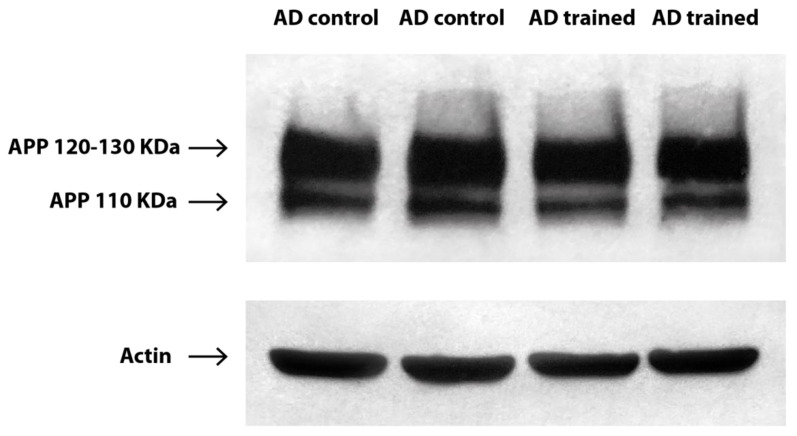
Western blots of APP and actin in platelet homogenates in the AD control and trained subjects at baseline. Samples were run in duplicate. AD trained subject is a 75-year-old woman with an MMSE of 22.3 and an APPr of 3.11. AD control subject is a 78-year-old man with an MMSE of 21.7 and an APPr of 2.94. The double band of APP at 120–130 kDa and 110 kDa is clearly visible. Actin was used as a loading control.

Baseline evaluation of patients assigned to the control group (*n* = 31) and the trained group (*n* = 28) established that the two cohorts were homogeneous for the clinical variables tested in the study, except for the forward verbal span test (Table 1 and Table 2). The effect of medications on the APPr was comparable in the two groups (data not shown). The Western blots of APP and actin, with the APP double band representing the 120–130 kDa and 110 kDa isoforms, are shown in Figure 1. The original images of Western blot are available online as Supplementary Materials.

### 2.2. Effects of the Cognitive Training Intervention

The effects of the cognitive training program were measured in each patient group as the difference (Δ) in test scores/values between each follow-up (FU) point and baseline; the Δ values computed for each FU point were then compared between the groups. As shown in Table 3, most significant differences between the groups were found at FU1 (at the end of the two-month intervention), where the trained group showed improved performances in several tasks, including the Instrumental Activities of Daily Living (IADL), the forward verbal span test, the backward verbal span test, the prose memory test, and the attentive matrices test. At FU2 (six months after the intervention), the significant differences involved the backward verbal span test, which improved in trained patients, and the Clinical Dementia Rating (CDR) score, which worsened in control patients. At FU3 (24 months after the intervention), significant differences were found only in the MMSE score and the APPr, but, strikingly, these reflected a worse condition in trained patients than in the control group (Table 3).

### 2.3. Associations between APPr and Clinical Variables

**Figure 2:**
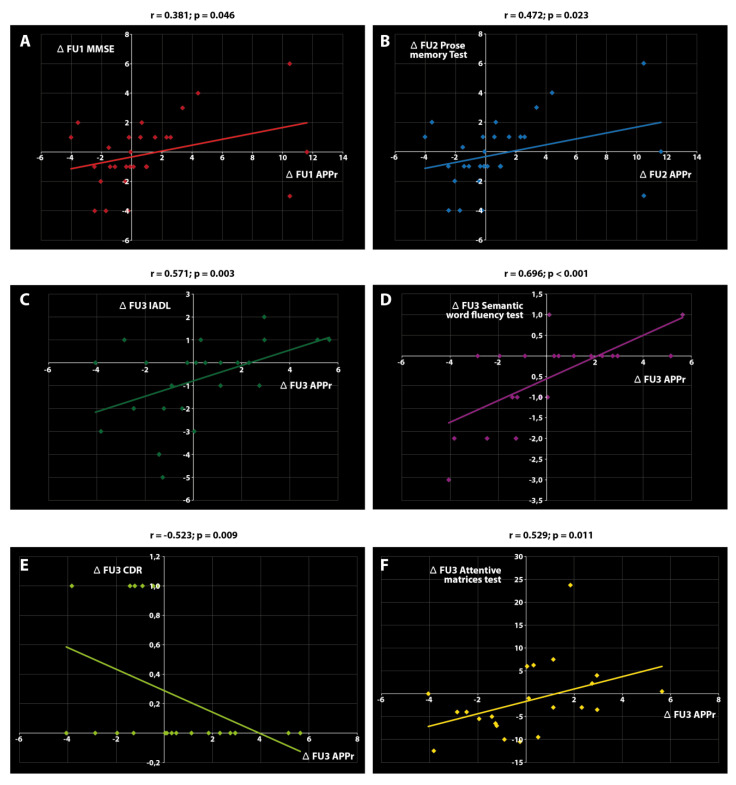
Significant correlations found in the control group. Δ APPr correlated with (**A**) Δ MMSE score at FU1, (**B**) Δ prose memory test at FU2, (**C**) Δ IADL at FU3, (**D**) Δ semantic word fluency test at FU3, (**E**) Δ CDR at FU3, and (**F**) Δ attentive matrices test at FU3. The correlation coefficient *r* and *p* values are reported for each diagram.

Analysis of the relationships between Δ APPr and the Δ scores of the other variables highlighted six significant correlations, all of which were found in the control group. In particular, Δ APPr correlated with (i) Δ MMSE at FU1, (ii) Δ prose memory test at FU2, and (iii) Δ IADL, Δ semantic word fluency test, Δ CDR, and Δ attentive matrices test at FU3 (Figure 2).

## 3. Discussion

The most striking findings of this study are that the cognitive training protocol induced a significant reduction in the platelet APPr 24 months after the intervention, coinciding with a significant reduction in MMSE scores, and that Δ APPr correlated with the Δ scores of some clinical variables (memory, attention, and executive function) exclusively in control patients. These data provide evidence of a complex relationship between platelet APPr and cognitive functions.

The APPr reduction and the significant reduction in MMSE scores found in trained patients two years after the intervention are surprising but in line with findings reported by our group in the same cohort [6]. Two other measures of dementia stage, namely, the Alzheimer’s Disease Assessment Scale-Cognitive Subscale and CDR, also showed the worst scores in the trained group at the last FU point, but differences regarding control patients were not significant. In contrast, some neuropsychological and cognitive variables, specifically those measuring memory and attention, increased significantly in trained vs. control patients at FU1 and FU2. These data suggest that cognitive training induced favorable short-term and adverse long-term effects in early-stage AD patients. Conceivably, the adverse long-term effects were due to the loss of homeostatic balance in these frail patients as a result of sustained alteration of their environmental conditions during cognitive training. Clearly, this hypothesis requires further investigation in larger patient samples using different cognitive training protocols. One of the possible perspectives is to associate personalized programs of cognitive training with the use of phytochemicals and botanicals with neuroprotective properties. Indeed, numerous works support that *Ginkgo biloba*, resveratrol, epigallocatechin- 3-gallate, and curcumin might have positive impacts on cognitive impairment [36].

While data analysis confirmed earlier reports of the correlation between the platelet APPr and MMSE and CDR scores [37,38,39], it also highlighted novel correlations with IADL, the prose memory test, the semantic word fluency test, and the attentive matrices test. Interestingly, these correlations were found exclusively in the control group. This finding can probably be ascribed to central and peripheral alterations in APP metabolism induced by cognitive training. APP plays multiple roles at synapses, such as axodentritic stabilization, synaptic vesicle processing, and preservation of memory cell substrates [40,41], and in structural dendritic spine plasticity, which encompasses morphological alterations at excitatory synapses [42]. APP processing changes occurring in the AD brain could therefore be mirrored by changes affecting platelets. Since the important role of APP in membrane anchoring and stability involves its effects on central synaptic activity, it is conceivable that cognitive training could alter the steady-state condition and abolish possible associations of the APPr with major clinical variables.

The demonstration that the platelet APPr correlates with key cognitive variables suggests a role for it as a peripheral biomarker of brain function in AD.

Why APP metabolism is altered in AD is an intriguing question. One explanation may be the accumulation throughout the body of cells with senescent phenotypes in subjects experiencing healthy aging, but particularly in those with age-related diseases. Senescent cells are characterized by metabolic dysregulation, mitochondrial dysfunction, altered proteostasis, and overexpression of pro-oxidant, proinflammatory, and matrix remodeling factors. Attrition of telomeres, the repetitive TTAGGG sequence found at chromosome ends, was identified as a major molecular determinant of senescence, involving cell cycle arrest in mitotic cells and aberrant cell cycle re-entry in postmitotic cells like neurons [43,44]. Regardless of the initiating events, a direct link between senescence and altered APP processing was demonstrated in human fibroblasts, where APP maturation was reduced in senescent cells and was directly mediated by senescence-associated increased membrane cholesterol [45]. In another model of human brain microvascular endothelial cells, senescence induced a reduction in APP expression that resulted in reduced secretion of the APP fragment generated by the cleavage of α-secretase (sAPPα), and increased expression of β-site APP cleaving enzyme (BACE1) and amyloid-β 40 production [46]. The influence of senescence on APP processing relates to altered proteostasis; through the disruption of proteome function and balance, senescence has the ability to affect protein synthesis, folding, quality control, and degradation rate, thereby producing misfolded proteins and aggregation of abnormal proteins, especially in AD [47]. Even though the best-known misfolded proteins in AD are amyloid-β and tau, our data suggest that other major targets, like APP, should be considered when devising therapeutic approaches or researching prognostic markers.

This study had some limitations that were identified via the lack of neuropathological confirmation of AD diagnosis, densitometric measurements of Western Blot bands as the sole experimental tool for APP isoform expression, and the statistical analysis of drug influence on platelet metabolism instead of treatment suspension before blood withdrawal, which need to be discussed. Lack of neuropathological confirmation is a problem in many AD research studies, and the possibility of misdiagnosis cannot be ruled out. However, we applied an extensive standardized workup, and all patients strictly fulfilled the established diagnostic criteria. Some papers verified dementia clinical diagnosis at autopsy, and neuropathology examination was reported to confirm clinical diagnosis in the majority of cases [48,49], thus strengthening the results of clinical diagnosis-based studies. APP isoform expression could also be evaluated by analyzing mRNA expression, with concomitant protein and mRNA expression analysis greatly enhancing the validity of the results [50]. Nevertheless, most studies rely exclusively on APP Western blot band measurements; a recent meta-analysis confirmed the close association of APPr with AD diagnosis [27]. In regard to drug influence on platelet metabolism, it should be pointed out that the majority of the studies on APPr used a cross-sectional design and the assumption of drugs known to affect platelet metabolism (anticoagulants, antiplatelet drugs, serotoninergic agonists–antagonists, and corticosteroids) was considered as an exclusion criterion [39,51,52]. The “My Mind Project” included AD patients that were followed for two years and it was not feasible to enroll AD patients not assuming any of these drugs. Therefore, all papers from this project relied on statistical analysis for the evaluation of drug influence on the biomarker under study.

## 4. Materials and Methods

### 4.1. Study Design and Participants

Participants were 59 patients with early AD participating in the “My Mind Project” [33]. Briefly, a diagnosis of probable early-stage AD was made based on a comprehensive clinical assessment that included neurological examination, neuropsychological evaluation, neuroimaging, and laboratory tests according to standard National Institute of Neurological and Communicative Disorders and Stroke–Alzheimer’s Disease and Related Disorders Association (NINCDS-ADRDA) diagnostic guidelines [53,54]. Sample size calculation was based on the hypothesis that there was a significant difference in APPr from baseline to FU2 after cognitive training in AD patients. Considering an effect size of 0.51 [30], a total sample of at least 53 subjects allowed 90% statistical power to be reached using Student’s t-test of matched pairs (difference between two dependent means) at the 5% level (two-tailed). The drop-out rate was set at 20%. The inclusion criteria comprised age ≥65 years, commitment to FU testing, diagnosis of mild-to-moderate dementia, and the presence of a caregiver. The exclusion criteria included deficits of the sensorimotor system, severe AD, neurodegenerative disorders other than AD, primary brain tumor, untreated epilepsy, major depression, schizophrenia, and alcohol or drug abuse over the past year. Patients meeting these criteria were randomly assigned to the control or the trained group. After baseline assessment of cognitive and neuropsychological performances and the collection of a peripheral blood sample for APPr determination, the control group received 2 months of general psychoeducational training on how to improve memory and health status, whereas the trained group received 2 months of comprehensive cognitive intervention. The program consisted of restorative cognitive training focusing on the enhancement of attentive functions, orientation, planning of activities of daily living, and episodic and prospective memory (see [55] for a full description). Cognitive and neuropsychological performance assessments and blood collection for APPr determination were again performed at the end of the psychoeducational training/cognitive intervention (FU1), and then at 6 (FU2) and 24 months (FU3). The study protocol complied with the principles of the Declaration of Helsinki and was approved by the local Ethics Committee (INRCA IRCCS Bioethics Advisory Committee, SC/12/301; 15 March 2012). Written informed consent was obtained from all participants or from their caregivers.

### 4.2. Western Blot Analysis and Calculation of the APP Ratio

A total amount of 7.5 mL of blood was collected by venipuncture in tubes containing 3.8% sodium citrate and 136 mM glucose (1:10 ratio with whole blood) at baseline and at each FU point. Blood samples were centrifuged at 200× *g* for 10 min to obtain platelet-rich plasma (PRP) as the supernatant fraction, which was separated by means of a plastic tip from the underlying buffy coat and erythrocytes. PRP was then centrifuged at 1500× *g* for 15 min to pellet the platelets. A total of 1.5 mL of Buffer B (10 mM Tris–HCl, 10 mM EDTA, 0.1 mM PMSF, 0.01 mg/L aprotinin, and 0.01 mg/L leupeptin, pH 7.4) was used to resuspend the platelets. After another centrifugation at 1500× *g* for 15 min, platelets were finally resuspended in 100 μL of Buffer B. Subsequently, platelets were subjected to 2 rounds of freeze–thawing (20 min each), sonicated for 20 s at 50% amplitude, and then stored at −80 °C until assayed. Platelet protein concentration was determined with the Lowry method. Following this, 30 µg of protein was separated on 7.5% precast polyacrylamide gels at 150 V for 40 min and electroblotted onto PVDF membranes using the Trans-Blot Turbo Transfer System, program Mixed MW (Bio-Rad, Hercules, CA, USA). Membranes were cut into two parts corresponding to high- and low-molecular weight proteins and were blocked with 5% non-fat dry milk overnight. Then, the part containing high molecular weight proteins was incubated for 1 h in a 1:1000 dilution of Anti-Alzheimer Precursor Protein A4, clone 22C11 (Merck, Darmstadt, Germany), which is specific for an epitope on the N-terminal portion of APP and detects several isoforms, including APP695, APP770, and APP751. The other part, containing low-molecular weight proteins, was exposed to Anti-Actin, clone C4 (Merck, Darmstadt, Germany). Membranes were then incubated in the secondary antibody, conjugated to horseradish peroxidase, and visualized using an enhanced chemiluminescence substrate (Bio-Rad, Hercules, CA, USA). Chemiluminescence detection was obtained with the ChemiDoc imaging system (Bio-Rad, Hercules, CA, USA), and quantitative analysis was performed by computer-assisted imaging (KS300 Imaging System, Zeiss, Oberkochen, Germany). Results were expressed as the ratio between the upper band (120–130 kDa, corresponding to APP770 and APP751) and the lower band (110 kDa, corresponding to APP695) densitometric units, using actin for data normalization. Briefly, an actin band was chosen as a reference and the ratio between the densitometric units of each actin band with the reference band was determined. Then, the two APP bands’ densitometric measures were multiplied by this ratio to obtain the normalized densitometric units for each subject.

### 4.3. Statistical Analysis

Before any analysis, the Kolmogorov–Smirnov test was applied to verify the normality of variable distribution. According to the test results, Student’s t-test was used to compare normally distributed variables while the Mann–Whitney test was employed to compare non-normally distributed variables. The χ^2^ test was applied to compare dichotomous variables. The baseline clinical test scores and the APPr of the two patient groups were compared. The use/non-use of drugs known to influence APP platelet metabolism was evaluated at baseline and at each FU point. Biomarker values were compared between users and non-users of acetylcholinesterase inhibitors, benzodiazepines, antidepressants, lipid-lowering medications, nonsteroidal anti-inflammatory drugs, anticoagulants, antihypertensives, antidiabetics, and corticosteroids. APPr values were compared in patients taking/not taking each medication. The effects of cognitive training were assessed by computing the FU and baseline scores/values of each variable in each group and by comparing their differences (Δ), i.e., increment or decrement, between the groups at each FU point. Any correlations between Δ APPr and the Δ scores of each neuropsychological and cognitive variable were evaluated separately in each group at each FU point using Spearman’s test with Bonferroni’s correction for multiple comparisons. A *p* value of < 0.05 was set for significance.

## Figures and Tables

**Table 1:** Characteristics of the study population divided by treatment.

	AD Control (*n* = 31)	AD Trained (*n* = 28)	*p*
Age (years)	78.74 ± 1.02	76.32 ± 0.86	0.078
Gender (females)	19 (61.29)	18 (64.28)	0.812
Schooling (years)	5.00 (3.00, 5.00)	5.00 (3.00, 5.00)	0.847
MMSE	20.70 ± 0.76	20.14 ± 0.86	0.627
GDS-30	8.06 ± 1.15	10.18 ± 1.33	0.231
ADL	6.00 (4.00, 6.00)	5.50 (5.00, 6.00)	0.622
IADL	4.00 (1.00, 5.00)	3.00 (2.00, 4.00)	0.639
PASE	61.22 ± 7.74	54.64 ± 7.15	0.537
ADAS-Cog	20.61 ± 1.85	20.39 ± 1.79	0.932
CDR	1.00 (1.00, 2.00)	1.00 (1.00, 2.00)	0.837
Age at onset (years)	76.52 ± 1.11	75.00 ± 0.87	0.294
Disease duration (months)	20.00 (4.00, 48.00)	10.00 (3.25, 28.75)	0.096
APPr	2.82 ± 0.30	3.38 ± 0.45	0.643

AD control = Alzheimer’s disease control group; AD trained = Alzheimer’s disease trained group; MMSE = Mini Mental State Examination; GDS-30 = Geriatric Depression Scale-30 questions; ADL = Activities of Daily Living; IADL = Instrumental Activities of Daily Living; PASE = Physical Activity Scale for the Elderly; ADAS-Cog = Alzheimer’s disease Assessment Scale-Cognitive Subscale; CDR = Clinical Dementia Rating; APPr = amyloid precursor protein ratio. Data are expressed as means ± SEM for normal continuous variables and as medians (Q1, Q3) for non-normal continuous variables. Gender is expressed as total number (%).

**Table 2:** Cognitive assessment of control and trained Alzheimer’s disease (AD) patients at baseline.

Test	AD Control (*n* = 31)	AD Trained (*n* = 28)	*p*
Forward verbal span	4.39 ± 0.14	3.88 ± 0.19	0.033
Backward verbal span	2.00 (2.00, 3.00)	2.00 (2.00, 2.00)	0.393
Prose memory	1.60 (1.00, 6.35)	3.38 (1.25, 7.33)	0.386
Word pairing learning	3.66 (2.50, 6.12)	3.86 (2.36, 5.86)	0.762
SWF	1.00 (0.00, 2.00)	1.00 (0.00, 2.00)	0.625
Attentive matrices	31.24 ± 1.78	27.26 ± 1.76	0.118
Corsi Supra Span	4.50 (3.50, 4.75)	4.13 (2.75, 5.31)	0.789

SWF = semantic word fluency. Data are expressed as means ± SEM for normal continuous variables and as medians (Q1, Q3) for non-normal continuous variables. Significant differences are in bold.

**Table 3:** Differences between follow-up and baseline scores/values of the clinical variables in control and treated patients with AD.

	Δ FU1		Δ FU2		Δ FU3	
	AD Control (*n* = 31)	AD Trained (*n* = 28)	AD Control (*n* = 31)	AD Trained (*n* = 28)	AD Control (*n* = 31)	AD Trained (*n* = 28)
**MMSE**	−0.14 ± 2.29	−0.63 ± 2.30	−1.42 ± 2.99	−1.92 ± 2.69	−1.95 ± 3.07	−4.75 ± 4.76 *
**GDS-30**	2.00 (−1.00, 4.00)	0.00 (−2.00, 3.00)	2.00 (−0.75, 4.75)	0.00 (−2.50, 3.50)	1.00 (−2.00, 3.00)	2.00 (−1.00, 4.00)
**ADL**	0.00 (0.00, 0.00)	0.00 (0.00, 0.00)	0.00 (−1.00, 0.00)	0.00 (−1.00, 0.00)	−1.00 (−2.00, 0.00)	−1.00 (−2.00, 0.00)
**IADL**	0.00 (−0.50, 0.00)	0.00 (0.00, 1.00) *	0.00 (−1.00, 0.00)	0.00 (−1.00, 1.00)	0.00 (−2.00, 0.00)	−1.00 (−3.00, 0.00)
**PASE**	16.27 ± 8.30	17.11 ± 7.75	−14.76 ± 7.94	−14.31 ± 5.42	−38.90 ± 8.39	−32.91 ± 7.69
**ADAS-Cog**	0.00 (−4.00, 3.50)	−3.00 (−6.00, −1.00)	0.00 (−4.00, 4.50)	−2.00 (−3.50, 1.50)	1.50 (−3.00, 6.00)	2.00 (−1.00, 23.00)
**CDR**	0.00 (0.00, 0.00)	0.00 (0.00, 0.00)	0.00 (0.00, 1.00)	0.00 (0.00, 0.00) *	0.00 (0.00, 1.00)	1.00 (0.00, 1.00)
**APPr**	1.01 ± 0.75	0.40 ± 0.72	−0.11 ± 0.47	−0.56 ± 0.50	0.25 ± 0.52	−1.79 ± 0.75 *
**Forward verbal span**	0.00 (−1.00, 1.00)	1.00 (0.00, 1.00) *	0.00 (−1.00, 1.00)	0.00 (−0.63, 1.00)	0.00 (−1.00, 0.43)	0.00 (−0.75, 0.83)
**Backward verbal span**	0.00 (−0.50, 0.00)	1.00 (0.00, 1.00) *	0.00 (−1.00, 0.00)	0.00 (0.00, 1.00) *	0.00 (−1.25, 0.00)	0.00 (−1.00, 0.00)
**Prose memory**	0.00 (−0.87, 0.43)	2.00 (0.00, 4.00) *	0.00 (−0.15, 1.83)	0.00 (−2.00, −2.55)	0.00 (−3.47, 1.83)	0.00 (−2.00, 2.00)
**Word pairing learning**	0.00 (−1.75, 1.01)	0.50 (−0.50, 2.00)	0.02 (−1.98, 1.52)	0.00 (−0.99, 1.75)	0.04 (−2.47, 1.79)	−0.47 (−1.97, 0.54)
**SWF**	0.00 (−0.50, 0.00)	0.00 (0.00, 0.00)	0.00 (0.00, 0.00)	0.00 (−0.50, 0.00)	0.00 (−1.00, 0.00)	−0.50 (−1.00, 0.00)
**Attentive matrices**	−1.00 (−2.25, 0.00)	2.00 (0.00, 5.00) *	0.00 (−3.50, 2.00)	2.00 (−6.00, 8.00)	−3.75 (−8.13, 0.38)	−4.00 (−6.00, 1.50)
**Corsi Supra Span**	0.00 (−1.00, 0.13)	0.00 (0.00, 1.00)	0.00 (−1.00, 0.00)	0.00 (−1.00, 1.00)	−1.00 (−1.87, 0.00)	−1.00 (−1.00, 0.00)

FU = follow-up; MMSE = Mini Mental State Examination; GDS-30 = Geriatric Depression Scale-30 questions; ADL = Activities of Daily Living; IADL = Instrumental Activities of Daily Living; PASE = Physical Activity Scale for the Elderly; ADAS-Cog = Alzheimer’s disease Assessment Scale-Cognitive Subscale; CDR = Clinical Dementia Rating; APPr = amyloid precursor protein ratio; SWF = semantic word fluency. Data are expressed as means ± SEM for normal continuous variables and as medians (Q1, Q3) for non-normal continuous variables. * *p* < 0.05, AD trained vs. AD control at the same FU point.

## Supplementary Materials

Supplementary materials can be found at https://www.mdpi.com/1422-0067/21/14/5110/s1.

## Abbreviations

AD	Alzheimer’s disease
APP	Amyloid precursor protein
APPr	Amyloid precursor protein ratio
FU1	Follow-up 1
FU2	Follow-up 2
FU3	Follow-up 3
MMSE	Mini Mental State Examination
IADL	Instrumental Activities of Daily Living
CDR	Clinical Dementia Rating
SEM	Standard Error of the Mean
sAPPα	Soluble amyloid precursor protein α
BACE1	β -secretase 1
PRP	Platelet-Rich Plasma
EDTA	Ethylenediaminetetraacetic acid
PMSF	Phenylmethylsulfonyl fluoride
PVDF	Polyvinylidene fluoride
