# Supplementary material for: Effect of a Cognitive Training Program on the Platelet APP Ratio in Patients with Alzheimer’s Disease
Source: Int J Mol Sci. 2020 Jul 20;21(14):5110. doi: 10.3390/ijms21145110 (PMC7403991; doi:10.3390/ijms21145110)
Supplement: Supplementary file 1 [file ijms-21-05110-s001.zip › Supporting information files/Explanatory file.docx]

**Supporting information**

The two TIFF files “IMG_6322” and “IMG_6323” represent the original images of Figure 1. More precisely the first four lanes from the left of “IMG_6322” and “IMG_6323” were used to show the Amyloid Precursor Protein and actin bands, respectively, in AD and control subjects.
