# Supplementary figures and images for: Effect of a Cognitive Training Program on the Platelet APP Ratio in Patients with Alzheimer’s Disease
Source: Int J Mol Sci. 2020 Jul 20;21(14):5110. doi: 10.3390/ijms21145110 (PMC7403991; doi:10.3390/ijms21145110)

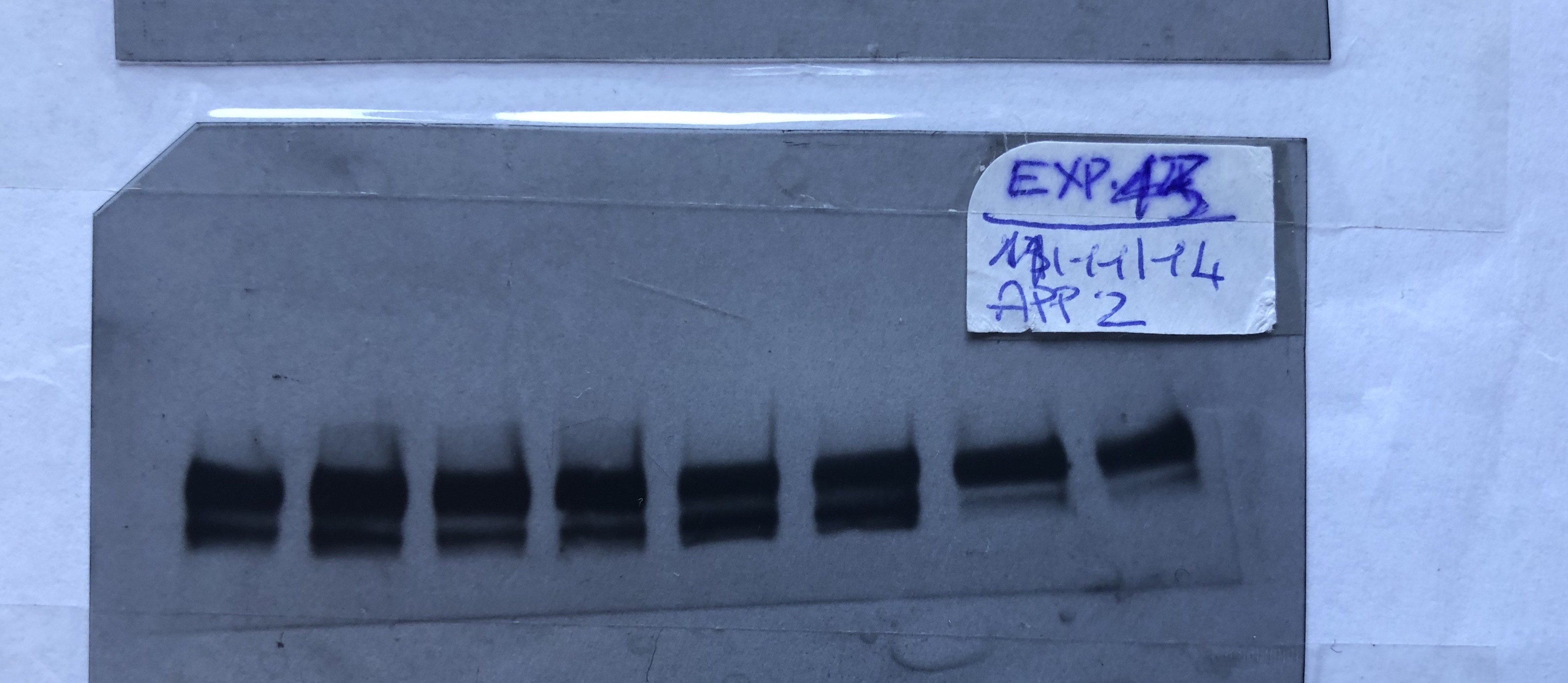

Supplement: Supplementary file 1 [file ijms-21-05110-s001.zip › Supporting information files/IMG_6322.tif]

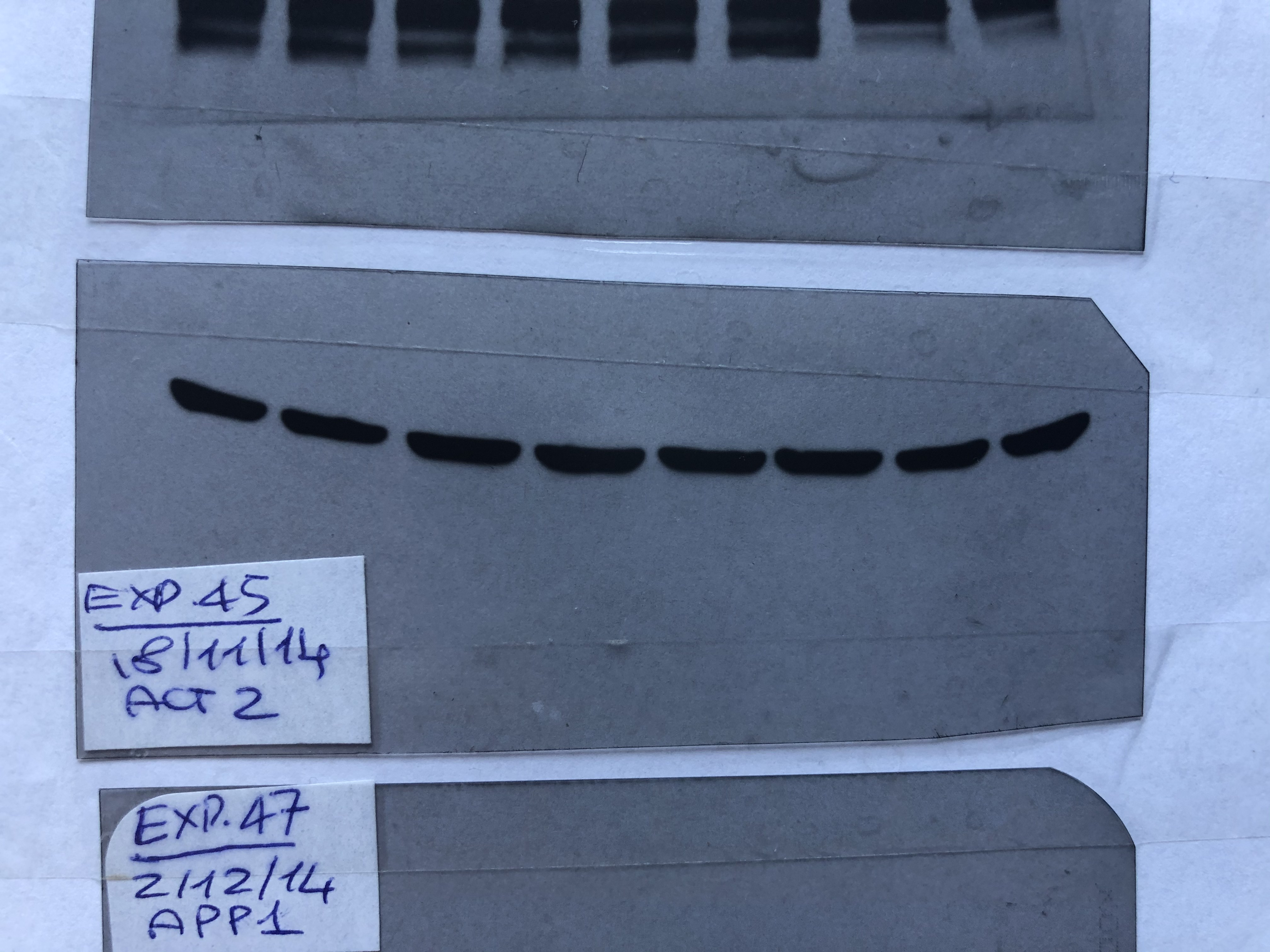

Supplement: Supplementary file 1 [file ijms-21-05110-s001.zip › Supporting information files/IMG_6323.tif]
